# Safety and Effectiveness of High‐Intensity Statins Versus Low/Moderate‐Intensity Statins Plus Ezetimibe in Patients With Atherosclerotic Cardiovascular Disease for Reaching LDL‐C Goals: A Systematic Review and Meta‐Analysis

**DOI:** 10.1002/clc.24334

**Published:** 2024-08-12

**Authors:** Hamidreza Soleimani, Asma Mousavi, Shayan Shojaei, Kiarash Tavakoli, Dorsa Salabat, Farid Farahani Rad, Mani K. Askari, John Nelson, Mohammed Ruzieh, Kaveh Hosseini

**Affiliations:** ^1^ Cardiac Primary Prevention Research Center, Cardiovascular Diseases Research Institute Tehran University of Medical Sciences Tehran Iran; ^2^ Tehran Heart Center, Cardiovascular Diseases Research Institute Tehran University of Medical Sciences Tehran Iran; ^3^ Department of Medicine University of Toledo Toledo Ohio USA; ^4^ California Cardiovascular Institute University of Louisville School of Medicine Fresno California USA; ^5^ Division of Cardiovascular Medicine, College of Medicine University of Florida, Gainsville Florida USA

**Keywords:** atherosclerosis, ezetimibe, meta‐analysis, statin, systematic review

## Abstract

**Background:**

It remains controversial whether adding ezetimibe to low/moderate‐intensity statins has a more beneficial impact on the treatment efficacy and safety of patients with existing atherosclerotic cardiovascular disease (ASCVD) compared to high‐intensity statin regimens.

**Hypothesis:**

A combination of low/moderate‐intensity statins plus ezetimibe might be more effective and safer than high‐intensity statin monotherapy.

**Methods:**

We searched databases for randomized controlled trials comparing lipid profile alterations, drug‐related adverse events, and MACE components between high‐intensity statin monotherapy and low/moderate‐intensity statin plus ezetimibe combination therapy. Pooled risk ratios (RR), mean differences (MD), and 95% confidence intervals (95% CI) were estimated using a random‐effects model.

**Results:**

Our comprehensive search resulted in 32 studies comprising 6162 patients treated with monotherapy against 5880 patients on combination therapy. Combination therapy was more effective in reducing low‐density lipoprotein cholesterol (LDL‐C) levels compared to monotherapy (MD = −6.6, 95% CI: −10.6 to −2.5); however, no significant differences were observed in other lipid parameters. Furthermore, the combination therapy group experienced a lower risk of myalgia (RR = 0.27, 95% CI: 0.13–0.57) and discontinuation due to adverse events (RR = 0.61, 95% CI: 0.51–0.74). The occurrence of MACE was similar between the two treatment groups.

**Conclusions:**

Adding ezetimibe to low/moderate‐intensity statins resulted in a greater reduction in LDL‐C levels, a lower rate of myalgia, and less drug discontinuation compared to high‐intensity statin monotherapy in patients with existing cardiovascular disease. However, according to our meta‐analysis, the observed reduction in LDL‐C levels in the combination group did not correlate with a reduction in MACE compared to the high‐intensity statin group.

## Summary

Combination therapy of low/moderate‐intensity statin with ezetimibe led to a greater reduction in LDL‐C levels, a lower rate of myalgia, and less drug discontinuation compared to high‐intensity statin monotherapy, but no significant effect on MACE components.

## Graphical abstract

Based on 32 RCTs, we compared the safety and effectiveness of combination therapy with ezetimibe and low/moderate‐intensity statin to high‐intensity statin monotherapy in patients with a history of atherosclerotic cardiovascular disease. The combination therapy demonstrated a greater LDL‐C reduction and fewer cases of myalgia and discontinuation due to drug‐related adverse events.

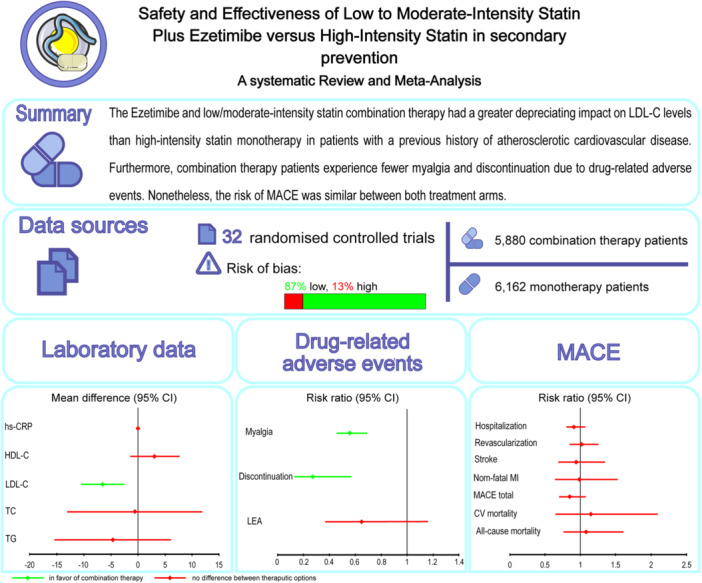

## 1 Introduction

Cardiovascular disease (CVD) is the leading cause of global mortality and disability, with elevated serum lipid levels, particularly low‐density lipoprotein cholesterol (LDL‐C) as one of its major risk factors [1, 2]. Lowering LDL‐C is crucial in mitigating the negative outcomes of CVD, especially in patients with a history of atherosclerotic cardiovascular disease (ASCVD). Statins are the first‐line therapy for patients with ASCVD for LDL‐C elevations, but many patients on low/moderate‐intensity statin regimens fail to attain target LDL‐C levels [3, 4]. Contemporary guidelines recommend high‐intensity statins for hyperlipidemia treatment, but they come with challenges such as drug interactions and dose‐dependent adverse events [5, 6]. An alternative approach is combining ezetimibe, a non‐statin drug, with tolerated stain dosage [7, 8]. Ezetimibe has shown efficacy in lowering LDL‐C alongside a more favorable safety profile [9, 10]. However, previous meta‐analyses have shown conflicting information regarding the superiority in efficacy or safety profile of high‐intensity statin monotherapy compared to low/moderate‐intensity statin combined with ezetimibe therapy [11, 12]. Given the recent randomized controlled trials (RCTs), we aimed to conduct a comprehensive systematic review and meta‐analysis comparing the efficacy and safety of these two lipid‐lowering strategies in ASCVD patients [13, 14, 15].

## 2 Materials and Methods

### 2.1 Protocol

We followed the Preferred Reporting Items for Systematic Reviews and Meta‐Analyses (PRISMA) guidelines to perform the study [16], and the study protocol was registered in the International Prospective Register for Systematic Reviews (PROSPERO) (registration number: CRD42023463729) [17].

### 2.2 Search Strategy and Study Selection

To identify relevant studies, we searched PubMed, Embase, and Web of Science databases from inception to July 2023. No language restrictions were applied. We also searched ClinicalTrials.gov and screened reference lists of eligible studies and relevant reviews. Detailed search terms are provided in Supporting Information S1: Table 1.

We included all RCTs comparing high‐intensity statin monotherapy with low/moderate‐intensity statin and ezetimibe combination therapy in ASCVD patients. We excluded conference abstracts, research letters, animal studies, non‐English publications, studies lacking RCT design, studies with the same dosage of statin in both arms, and studies without full text. For studies with multiple publications, we included the publication with the most detailed information.

### 2.3 Risk of Bias Assessment

Two authors (A.M. and S.S.) independently screened titles and abstracts, and discrepancies were resolved through discussion with senior authors (K.H. and H.S.). The risk of bias of the included studies was assessed using RoB2 instructions, with discrepancies resolved by consulting the senior authors [18]. Results are provided in Supporting Information S1: Table 2.

### 2.4 Data Extraction

We extracted relevant data including author, publication year, study location, statin type and dose, treatment duration, sample sizes, demographics (including age, sex, background disease, and cardiovascular risk factors including hypertension and diabetes), and outcome data (serum lipid profile including LDL‐C, high‐density lipoprotein cholesterol [HDL‐C], total cholesterol [TC], and triglyceride [TG]; high‐sensitivity C‐reactive protein [hs‐CRP] biomarker; drug‐related adverse events such as elevation of liver enzymes, myalgia, discontinuation due to adverse events; and composite MACE and its components including hospitalization, revascularization, non‐fatal myocardial infarction [MI], stroke, all‐cause mortality, and cardiovascular mortality).

### 2.5 Data Preparation and Statistical Analyses

For analysis, we calculated pooled risk ratios (RR), mean differences (MD), and 95% confidence intervals (95% CIs) using a random‐effects model. Heterogeneity was assessed using *I* ^2^ statistics, and subgroup analyses were conducted based on statin type and treatment duration to find the source of heterogeneity. Publication bias was assessed using funnel plots and Egger's regression (if ≥ 10 studies). Analyses were performed using R (version 4.1.3 for Windows, Vienna, Austria) and R studio (version 1.1.463, Posit PBC, Boston, MA) with “tidyverse” and “meta” packages.

## 3 Results

**Figure 1:**
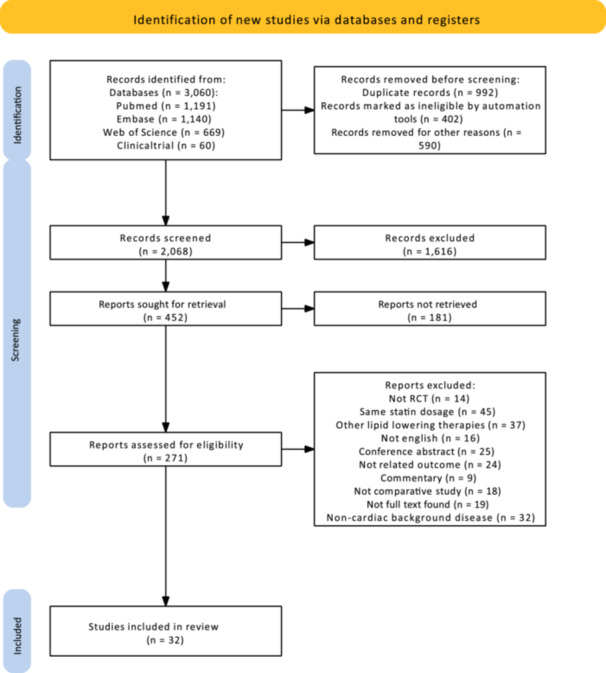
PRISMA flow chart.

The search strategy yielded a total of 3060 articles. After removing duplicates and screening of articles, 32 trials were included in the meta‐analysis (Figure 1). Inter‐reader agreement was high (*κ* coefficient = 0.9).

The included articles consisted of 12 042 ASCVD patients, among which 5880 (48.42%) participants received low/moderate‐intensity statin with ezetimibe, whereas 6162 participants received high‐intensity statin monotherapy. The included studies employed various statins, namely rosuvastatin (*n* = 5) [13, 19, 20, 21, 22], atorvastatin (*n* = 12) [15, 23, 24, 25, 26, 27, 28, 29, 30, 31, 32, 33], simvastatin (*n* = 3) [34, 35, 36], pravastatin (*n* = 1) [37], multiple statins (*n* = 10) [7, 38, 39, 40, 41, 42, 43, 44, 45], and one study used both atorvastatin and multiple statins [46]. Approximately half of the studies were blinded (47%), whereas blindness was not mentioned in 40% of studies. Only two studies used a placebo in the monotherapy arm [32, 34]. The duration of treatment in these studies ranged from 1 to 36 months from baseline. Supporting Information S1: Table 3 provides an overview of the key characteristics of the included studies.

### 3.1 Laboratory Data

**Figure 2:**
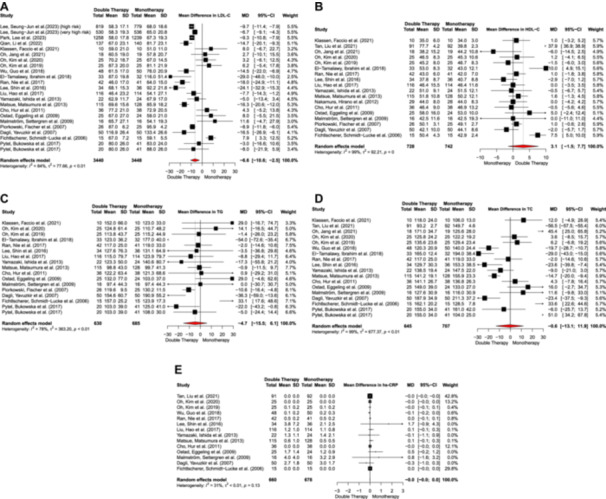
(A) Mean difference in LDL‐C, (B) mean difference in HDL‐C, (C) mean difference in TG, (D) mean difference in TC, and (E) mean difference in hs‐CRP.

Twenty‐one studies compared high‐intensity statin monotherapy with low/moderate‐intensity statin with ezetimibe combination therapy on LDL‐C levels in ASCVD patients. The combination therapy demonstrated a greater reduction in LDL‐C levels compared to monotherapy (MD = −6.6, 95% CI: −10.6 to −2.5). However, a high degree of heterogeneity was noted among the included studies (*I* ^2^ = 84%, *p* < 0.01) (Figure 2A). The preliminary inspection of the funnel plot and Egger's regression showed no statistical evidence of publication bias (Supporting Information S1: Figure 1A). The observed effects remained consistent when considering treatment durations of less than 12 months or more than 12 months (Supporting Information S1: Figure 2A). The reduction in LDL‐C levels was exclusively observed in atorvastatin and rosuvastatin subgroups (*p* < 0.001) (Supporting Information S1: Figure 2B). Considering different subgroups, the heterogeneity of effect was not due to variations in used statins or treatment durations across the included studies.

**Figure 3:**
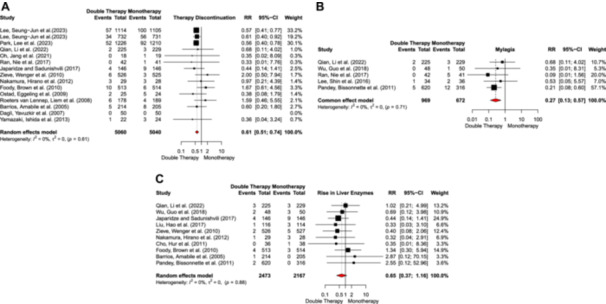
(A) Therapy discontinuation, (B) myalgia, and (C) rise in liver enzymes.

**Figure 4:**
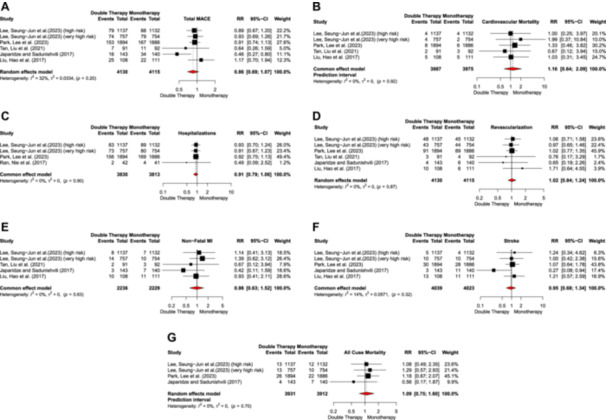
(A) Total MACE, (B) cardiovascular mortality, (C) hospitalization, (D) revascularization, (E) non‐fetal MI, (F) stroke, and (G) all‐cause mortality.

The difference between the effect of high‐intensity statin monotherapy and low/moderate‐intensity statin with ezetimibe combination therapy on HDL‐C, TG, TC, and hs‐CRP was evaluated in 18, 16, 17, and 14 studies, respectively. All of the analyses demonstrated no significant difference in terms of HDL‐C (MD = 3.1, 95% CI: −1.5 to 7.7), TG (MD = −4.7, 95% CI: −16.5 to 6.1), TC (MD = −0.6, 95% CI: −13.1 to 11.9), and hs‐CRP (MD = 0, 95% CI: −0.0 to 0.0) levels between the treatment arms. Considerable heterogeneity was noted among the studies, with *I* ^2^ values of 99% (*p* = 0) for HDL‐C, 78% (*p* < 0.01) for TG, 99% (*p* < 0.01) for TC, and 31% (*p* = 0.13) for hs‐CRP (Figure 2B–E). Although no significant publication bias was observed for HDL‐C, TG, and hs‐CRP, the inspection of the funnel plot and Egger's test demonstrated the existence of publication bias for TC (Supporting Information S1: Figure 1B–E). Subgroup analyses based on the statin class (Supporting Information S1: Figures 3B, 4B, 5B, and 6B) and treatment duration (Supporting Information S1: Figures 3A, 4A, 5A, and 6A) did not reveal any significant differences between the efficacy of the two treatment arms. Among the studies analyzing HDL‐C subgroups, heterogeneity was attributed to the atorvastatin subgroup. In the case of TC, heterogeneity was influenced by studies involving multiple statins and atorvastatin. Subgroup analyses of different statins used and treatment periods are summarized in Supporting Information S1: Table 4.

### 3.2 Drug‐Related Adverse Events

Discontinuation due to adverse events in both treatment arms was evaluated in 14 studies. The risk of discontinuation due to adverse events was significantly lower in the combination therapy group compared to the monotherapy treatment arm (RR = 0.61, 95% CI: 0.51–0.74). The studies showed low heterogeneity (*I* ^2^ = 0%, *p* = 0.61) (Figure 3A). Subgroup analysis based on drug type demonstrated that the observed effect was exclusive to the Rosuvastatin subgroup, whereas no significant differences were observed in others (*p* = 0.04) (Supporting Information S1: Figure 7B). Interestingly, further subgroup analysis of the included studies demonstrated that the risk of discontinuation due to adverse events was approximately twofold in the monotherapy arm after 12 months of treatment (Supporting Information S1: Figure 7A). Examining other adverse events, the analysis indicated a lower risk of myalgia in the combination therapy group compared to the monotherapy arm (RR = 0.27, 95% CI: 0.13–0.57) based on the findings of five studies. Conversely, there was no significant difference between the two treatment modalities regarding liver enzyme elevations based on 10 included studies (RR = 0.65, 95% CI: 0.37–1.16). No heterogeneity was observed among studies in both parameters (Figure 3B,C). Additionally, no significant publication bias was observed for discontinuation due to adverse events and liver enzyme elevations (Supporting Information S1: Figure 8A,B). Subgroup analyses of the different statins used are presented in Supporting Information S1: Table 5.

### 3.3 MACE

A total of five studies examined the effect of low/moderate‐intensity statin with ezetimibe combination therapy on composite MACE compared to high‐intensity statin monotherapy. The analysis did not reveal significant differences between the treatment groups (RR = 0.86, 95% CI: 0.69–1.07) (Figure 4A). The use of ezetimibe plus low/moderate‐intensity statin compared to high‐intensity statin alone had no significant effect on the individual component of MACE, including cardiovascular mortality (RR = 1.16, 95% CI: 0.64–2.09; Figure 4B), hospitalization (RR = 0.91, 95% CI: 0.79–1.06; Figure 4C), revascularization (RR = 1.02, 95% CI: 0.84–1.24; Figure 4D), non‐fatal MI (RR = 0.98, 95% CI: 0.63–1.52; Figure 4E), stroke (RR = 0.95, 95% CI: 0.68–1.34; Figure 4F), or all‐cause mortality (RR = 1.09, 95% CI: 0.75–1.60; Figure 4G). Heterogeneity among the studies for each parameter was reported to be low.

## 4 Discussion

Approximately 25% of high‐risk and very high‐risk ASCVD patients achieve the LDL‐C goals recommended by the 2019 ECS/EAS guidelines [47, 48]. Although ezetimibe plus low/moderate‐intensity statin therapy has been shown to reduce CVD event rates and increase LDL‐C goal attainment, its utilization remains low. In a recent study, only 4% of high‐risk and 9% of very high‐risk patients were on combination therapy [49]. Furthermore, ezetimibe combined with low/moderate‐intensity statin is available, affordable, and cost‐effective [50]. Our study has significant implications for ezetimibe in LDL‐C goal attainment. In the present study, we showed that low/moderate‐intensity statin with ezetimibe combination therapy has a greater depreciating impact on LDL‐C levels compared to high‐intensity statin monotherapy in ASCVD patients. Moreover, we found that patients undergoing combination therapy experience fewer drug‐related myalgia and discontinuation due to adverse events. Nevertheless, the occurrence of MACE between the two treatment groups was similar despite a more pronounced decrease in LDL‐C levels in the combination therapy arm.

Although previous studies have examined the efficacy and safety of these therapies, to our knowledge, this is the most comprehensive systematic review and meta‐analysis comparing the efficacy of these two treatment approaches on lipid and hs‐CRP biomarkers, adverse events, and MACE components altogether. Our study supports the increased utilization of ezetimibe to improve LDL‐C guideline goal attainment.

### 4.1 Laboratory Data

Our findings demonstrated that the combination therapy approach was more effective in reducing LDL‐C levels compared to statin monotherapy; however, no significant difference was noted in other lipid parameters. Our results aligned with a previous meta‐analysis study conducted by Ah et al., which reviewed all studies until 2021 [11]. Their study, which included 18 publications, showed that adopting low/moderate‐intensity statin plus ezetimibe was more effective in reducing not only LDL‐C but also other lipid parameters and hs‐CRP. However, their data were older, and many recent studies were omitted from the analysis, especially those published in the last 2 years. In contrast to the aforementioned systematic review, our study focuses exclusively on patients with a history of CVD to minimize the confounding effect of non‐cardiovascular conditions such as metabolic syndrome, diabetes, and hypercholesterolemia on outcomes. Furthermore, unlike the previous study, we conducted a subgroup analysis to investigate the effect of low/moderate‐intensity statin plus ezetimibe therapy in different statin types and treatment durations.

The meta‐analysis by Zhu et al. published in 2020 compared high‐intensity statin monotherapy with low/moderate‐intensity statin and ezetimibe combination therapy in patients with high cardiovascular risk [12]. Consistent with the present study, it reported that the combination therapy was more efficient in reaching target LDL‐C levels compared to statin monotherapy by including 14 studies. However, in contrast to our findings, a greater reduction in non‐HDL and TC levels was observed in the combination therapy group. The findings of this meta‐analysis have been updated through our meta‐analysis by including a larger number of recent studies. Additionally, the results of the aforementioned study were limited to treatment durations under 1 year, whereas our study demonstrated that the benefits of combination therapy on LDL‐C levels persist even in longer treatment durations. It is worth noting that LDL‐C reduction is the primary treatment goal for patients with CVD. By combining a low/moderate‐intensity statin with ezetimibe, clinicians can achieve more LDL‐C reduction, which may translate to improved long‐term cardiovascular outcomes. However, the lack of significant difference in other lipid parameters suggests that the benefits of combination therapy may be primarily driven by the enhanced LDL‐C lowering effect. One reason for this observation is that the regulation of LDL‐C is more directly linked to the cholesterol biosynthesis pathway inhibited by statins [51]. Additionally, patients with different baseline lipid abnormalities may respond differently to statin therapy. For example, individuals with predominantly elevated LDL‐C may experience more robust LDL‐C reduction, whereas those with hypertriglyceridemia may have more variable effects on TG levels [52].

### 4.2 Drug‐Related Adverse Event

Our analysis suggested that a combination of low/moderate‐intensity statin and ezetimibe carries lower risks of discontinuation due to adverse events and myalgia compared to high‐intensity statin monotherapy. In contrast to our findings, Zhan et al. found no difference in regard to treatment discontinuation due to adverse events by evaluating 10 RCTs [53]. However, their results were mainly based on low‐quality studies, and did not contain more recent publications. Chaiyasothi et al. found no difference in treatment discontinuation due to drug‐related adverse events [54], but they comprised both moderate and high‐intensity statin in the monotherapy group.

In our study, the risk of elevated liver enzymes was comparable between the two treatment groups. Previous meta‐analyses also demonstrated that there was no significant difference in terms of elevated liver enzymes in both treatment methods [11, 12, 53]. Nevertheless, it's worth noting that these earlier meta‐analyses did not include more recent RCTs with longer treatment durations. It is essential to note that treatment adherence and persistence are crucial for achieving long‐term cardiovascular benefits. The improved tolerability of the combination therapy may lead to better patient compliance and, ultimately, improved clinical outcomes. The comparable risk of elevated liver enzymes between the two treatment groups is also reassuring, as this adverse event is a common concern with statin use.

### 4.3 MACE

We did not find a meaningful difference in MACE between the two treatment methods. The results of the meta‐analysis conducted by Zhan et al. in 2018 also corroborate comparable all‐cause mortality and cardiovascular mortality between the two treatment groups. However, patients have a lower risk of non‐fatal MI and stroke in the combination therapy group [53]. The findings of the cohort study conducted by Kim et al. were also in agreement with our finding by showing that there was no significant difference in the risk of non‐fatal MI, repeat revascularization, stroke, and all‐cause mortality between two treatment groups after 5‐year follow‐up [13, 14].

There are two main hypotheses to elucidate the conflicting outcomes. First, most analyses on MACE outcomes didn't comprise patients who discontinued their treatment due to adverse events and were more susceptible to cardiovascular outcomes [13, 14, 19, 20, 21, 22]. Second, the different dosages and types of statins used in various therapies among the related studies could be an underlying cause of these conflicting results. Although our study did not demonstrate a clear superiority of combination therapy in reducing MACE, the significant reduction in LDL‐C levels and improved tolerability profiles observed with this approach may still provide important clinical benefits. Clinicians should consider these factors when weighing the risks and benefits of statin monotherapy versus combination therapy for individual patients.

## 5 Limitations

The primary limitation of our study is the inherent heterogeneity among the included studies. Variations in statin classifications, dosages, and follow‐up durations may have contributed to the heterogeneities observed. Additionally, because this was a study‐level meta‐analysis lacking patient‐level data, we were unable to perform more in‐depth subgroup analyses or meta‐regressions. The second limitation is due to the publication bias. Despite our efforts to minimize publication bias by conducting a comprehensive search and including unpublished studies, the possibility of bias can't be completely ruled out. In addition, our outcomes for TC have been influenced by publication bias and, therefore, needed to be interpreted with caution. It is worth noting that publication bias was not assessed for some of our outcome parameters, as Egger's test and funnel plots are not appropriate in analyses containing less than 10 studies. The third limitation was about limited assessment of adverse events and MACE. Although we assessed the MACE components in both treatment groups, it is important to note that our analysis may have been limited by the availability of data reported in the included studies. Although most of the RCTs in our analysis were blinded, the types of blindness were not mentioned in these studies. Variations in the definitions and reporting of adverse events and MACE components across studies may have affected the accuracy and comparability of the data. It would be nice if our findings were supported by large‐scale, blinded RCTs with longer follow‐up durations to better evaluate the long‐term impact of statin plus ezetimibe combination therapy on hard cardiovascular events, patient‐reported quality of life, and treatment satisfaction to better understand the real‐world impact of the two treatment strategies on the overall well‐being of ASCVD patients.

## 6 Conclusions

In conclusion, this comprehensive systematic review and meta‐analysis evaluated the efficacy and safety of the combination therapy versus monotherapy in terms of lipid levels, hs‐CRP biomarkers, adverse events, and MACE in patients with existing CVD. Based upon the overall analysis of our study, the combination therapy of low/moderate‐intensity statins with ezetimibe led to a greater reduction in LDL‐C levels, a lower rate of myalgia, and less drug discontinuation compared to high‐intensity statin monotherapy, but no significant effect on MACE components.

## Author Contributions

Conceptualization: Kaveh Hosseini. Methodology: Asma Mousavi, Shayan Shojaei, Dorsa Salabat, and Farid Farahani Rad. Formal analysis and investigation: Hamidreza Soleimani. Writing–original draft preparation: Asma Mousavi, Shayan Shojaei, Kiarash Tavakoli, Dorsa Salabat, and Farid Farahani Rad. Writing–review and editing: Hamidreza Soleimani, Mani K. Askari, John Nelson, Mohammed Ruzieh, and Kaveh Hosseini. Supervision: Kaveh Hosseini. All authors read and approved the final manuscript.

## Use of AI and AI‐assisted Technologies Statement

It has not been used.

## Disclosure

The authors have nothing to report.

## Conflicts of Interest

The authors declare no conflicts of interest.

## Supporting information

Supporting information.

## Data Availability

The data that support the findings of this study are available in the Supporting Information material of this article.

## References

[1] G. A. Roth , G. A. Mensah , C. O. Johnson , et al., “Global Burden of Cardiovascular Diseases and Risk Factors, 1990–2019,” Journal of the American College of Cardiology 76, no. 25 (2020): 2982–3021. 33309175 10.1016/j.jacc.2020.11.010 PMC7755038

[2] H. Du , Q. Shi , P. Song , et al., “Global Burden Attributable to High Low‐Density Lipoprotein‐Cholesterol From 1990 to 2019,” Frontiers in Cardiovascular Medicine 9 (2022): 903126. 35757342 10.3389/fcvm.2022.903126 PMC9218272

[3] D. K. Arnett , R. S. Blumenthal , M. A. Albert , et al., “2019 ACC/AHA Guideline on the Primary Prevention of Cardiovascular Disease: Executive Summary: A Report of the American College of Cardiology/American Heart Association Task Force on Clinical Practice Guidelines,” Circulation 140, no. 11 (2019): e563–e595. 30879339 10.1161/CIR.0000000000000677 PMC8351755

[4] K. Wilemon , D. MacDougall , M. McGowan , W. Howard , and K. Myers , “71% of High Risk Hypercholesterolemia Patients Never Reach ACC AHA Guidelines,” supplement, Journal of the American College of Cardiology, 81, no. 8_Suppl (2023): 1231.36990541

[5] S. M. Grundy and N. J. Stone , “2018 American Heart Association/American College of Cardiology/Multisociety Guideline on the Management of Blood Cholesterol–Secondary Prevention,” JAMA Cardiology 4, no. 6 (2019): 589–591. 30994869 10.1001/jamacardio.2019.0911

[6] P. D. Thompson , G. Panza , A. Zaleski , and B. Taylor , “Statin‐Associated Side Effects,” Journal of the American College of Cardiology 67, no. 20 (2016): 2395–2410. 27199064 10.1016/j.jacc.2016.02.071

[7] J. M. Foody , W. V. Brown , F. Zieve , et al., “Safety and Efficacy of Ezetimibe/Simvastatin Combination Versus Atorvastatin Alone in Adults ≥ 65 Years of Age With Hypercholesterolemia and With or at Moderately High/High Risk for Coronary Heart Disease (The VYTELD Study),” The American Journal of Cardiology 106, no. 9 (2010): 1255–1263. 21029821 10.1016/j.amjcard.2010.06.051

[8] S. V. Rao , H. R. Reynolds , and J. S. Hochman , “Chronic Coronary Disease Guidelines,” Circulation 148, no. 9 (2023): 729–731. 37471475 10.1161/CIRCULATIONAHA.123.064623

[9] Y. Wang , S. Zhan , H. Du , et al., “Safety of Ezetimibe in Lipid‐Lowering Treatment: Systematic Review and Meta‐Analysis of Randomised Controlled Trials and Cohort Studies,” BMJ Medicine 1, no. 1 (2022): e000134. 36936552 10.1136/bmjmed-2022-000134 PMC10012858

[10] J. Underberg , P. P. Toth , and F. Rodriguez , “LDL‐C Target Attainment in Secondary Prevention of ASCVD in the United States: Barriers, Consequences of Nonachievement, and Strategies to Reach Goals,” Postgraduate Medicine 134, no. 8 (2022): 752–762. 36004573 10.1080/00325481.2022.2117498

[11] Y.‐M. Ah , M. Jeong , and H. D. Choi , “Comparative Safety and Efficacy of Low‐or Moderate‐Intensity Statin Plus Ezetimibe Combination Therapy and High‐Intensity Statin Monotherapy: A Meta‐Analysis of Randomized Controlled Studies,” PLoS One 17, no. 3 (2022): e0264437. 35245303 10.1371/journal.pone.0264437 PMC8896700

[12] Y. Zhu , H. Hu , J. Yang , et al., “The Efficacy and Safety of Statin in Combination With Ezetimibe Compared With Double‐Dose Statin in Patients With High Cardiovascular Risk: A Meta‐Analysis,” Bosnian Journal of Basic Medical Sciences 20, no. 2 (2020): 169–182. 31668143 10.17305/bjbms.2019.4437 PMC7202188

[13] S.‐H. Lee , Y.‐J. Lee , J. H. Heo , et al., “Combination Moderate‐Intensity Statin and Ezetimibe Therapy for Elderly Patients With Atherosclerosis,” Journal of the American College of Cardiology 81, no. 14 (2023): 1339–1349. 37019580 10.1016/j.jacc.2023.02.007

[14] K. Kim , W.‐D. Bang , K. Han , B. Kim , J. M. Lee , and H. Chung , “Comparison of the Effects of High‐Intensity Statin Therapy With Moderate‐Intensity Statin and Ezetimibe Combination Therapy on Major Adverse Cardiovascular Events in Patients With Acute Myocardial Infarction: A Nationwide Cohort Study,” Journal of Lipid and Atherosclerosis 10, no. 3 (2021): 291. 34621700 10.12997/jla.2021.10.3.291 PMC8473958

[15] P. C. Oh , A. Y. Jang , K. Ha , et al., “Effect of Atorvastatin (10 mg) and Ezetimibe (10 mg) Combination Compared to Atorvastatin (40 mg) Alone on Coronary Atherosclerosis,” The American Journal of Cardiology 154 (2021): 22–28. 34238445 10.1016/j.amjcard.2021.05.039

[16] M. J. Page , J. E. McKenzie , P. M. Bossuyt , et al., “The PRISMA 2020 Statement: An Updated Guideline for Reporting Systematic Reviews,” Systematic Reviews 10 (2021): 89. 33781348 10.1186/s13643-021-01626-4 PMC8008539

[17] ”Assessing Safety of SGLT2i Drugs Among Heart Failure Patients: A Systematic Review and Meta‐Analysis,” PROSPERO, accessed June 20, 2023, https://www.crd.york.ac.uk/prospero/display_record.php?ID=CRD42023424911.

[18] J. P. Higgins and D. G. Altman , “Assessing Risk of Bias in Included Studies,” in Cochrane Handbook For Systematic Reviews of Interventions: Cochrane Book Series, eds. J. P. Higgins and S. Green (Chichester: Wiley, 2008), 187–241.

[19] M. Oh , H. Kim , E. W. Shin , et al., “Comparison of High‐Dose Rosuvastatin Versus Low‐Dose Rosuvastatin Plus Ezetimibe on Carotid Atherosclerotic Plaque Inflammation in Patients With Acute Coronary Syndrome,” Journal of Cardiovascular Translational Research 13 (2020): 900–907. 32367340 10.1007/s12265-020-10009-4

[20] D. Ran , H. Nie , Y. Gao , et al., “A Randomized, Controlled Comparison of Different Intensive Lipid‐Lowering Therapies in Chinese Patients With Non‐ST‐Elevation Acute Coronary Syndrome (NSTE‐ACS): Ezetimibe and Rosuvastatin Versus High‐Dose Rosuvastatin,” International Journal of Cardiology 235 (2017): 49–55. 28291622 10.1016/j.ijcard.2017.02.099

[21] J.‐I. Park , S.‐J. Lee , and B.‐K. Hong , et al., “Efficacy and Safety of Moderate‐Intensity Statin With Ezetimibe Combination Therapy in Patients After Percutaneous Coronary Intervention: A Post‐Hoc Analysis of the RACING Trial,” Eclinicalmedicine 58 (2023): 101933. 37090440 10.1016/j.eclinm.2023.101933 PMC10119495

[22] D. Yamazaki , M. Ishida , H. Watanabe , et al., “Comparison of Anti‐Inflammatory Effects and High‐Density Lipoprotein Cholesterol Levels Between Therapy With Quadruple‐Dose Rosuvastatin and Rosuvastatin Combined With Ezetimibe,” Lipids in Health and Disease 12 (2013): 9. 23374898 10.1186/1476-511X-12-9 PMC3598241

[23] M. M. El‐Tamalawy , O. M. Ibrahim , T. M. Hassan , and A. A. El‐Barbari , “Effect of Combination Therapy of Ezetimibe and Atorvastatin on Remnant Lipoprotein Versus Double Atorvastatin Dose in Egyptian Diabetic Patients,” The Journal of Clinical Pharmacology 58, no. 1 (2018): 34–41. 28858387 10.1002/jcph.976

[24] Y. Matsue , A. Matsumura , M. Suzuki , Y. Hashimoto , and M. Yoshida , “Differences in Action of Atorvastatin and Ezetimibe in Lowering Low‐Density Lipoprotein Cholesterol and Effect on Endothelial Function–Randomized Controlled Trial,” Circulation Journal 77, no. 7 (2013): 1791–1798. 23603824 10.1253/circj.cj-13-0033

[25] Z. Liu , H. Hao , C. Yin , Y. Chu , J. Li , and D. Xu , “Therapeutic Effects of Atorvastatin and Ezetimibe Compared With Double‐Dose Atorvastatin in Very Elderly Patients With Acute Coronary Syndrome,” Oncotarget 8, no. 25 (2017): 41582–41589. 28177908 10.18632/oncotarget.15078 PMC5522285

[26] L. Japaridze and M. Sadunishvili , “The Short‐Term Effect of Atorvastatin Plus Ezetimibe Therapy Versus Atorvastatin Monotherapy on Clinical Outcome in Acute Coronary Syndrome Patients by Gender,” Kardiologia Polska 75, no. 8 (2017): 770–778. 28553847 10.5603/KP.a2017.0074

[27] M. A. Ostad , S. Eggeling , P. Tschentscher , et al., “Flow‐Mediated Dilation in Patients With Coronary Artery Disease Is Enhanced by High Dose Atorvastatin Compared to Combined Low Dose Atorvastatin and Ezetimibe: Results of the CEZAR Study,” Atherosclerosis 205, no. 1 (2009): 227–232. 19150064 10.1016/j.atherosclerosis.2008.11.032

[28] J. T. Palathingal , D. Vijayan , D. Rajan C , I. Stanly , T. Jamshad , and M. Linu , “A Randomised Controlled Study of High Dose Statin Versus Statin Plus Ezetimibe Therapy in Patients With Acute Coronary Syndrome,” International Journal of Biomedical Science: IJBS 16 (2020): 52–67.

[29] M. Piorkowski , S. Fischer , C. Stellbaum , et al., “Treatment With Ezetimibe Plus Low‐Dose Atorvastatin Compared With Higher‐Dose Atorvastatin Alone,” Journal of the American College of Cardiology 49, no. 10 (2007): 1035–1042. 17349882 10.1016/j.jacc.2006.10.064

[30] J. Qian , Z. Li , X. Zhang , et al., “Efficacy and Tolerability of Ezetimibe/Atorvastatin Fixed‐Dose Combination Versus Atorvastatin Monotherapy in Hypercholesterolemia: A Phase III, Randomized, Active‐Controlled Study in Chinese Patients,” Clinical Therapeutics 44, no. 10 (2022): 1282–1296. 36182594 10.1016/j.clinthera.2022.08.013

[31] H. Tan , L. Liu , Q. Zheng , et al., “Effects of Combined Lipid‐Lowering Therapy on Low‐Density Lipoprotein Cholesterol Variability and Cardiovascular Adverse Events in Patients With Acute Coronary Syndrome,” Advances in Therapy 38 (2021): 3389–3398. 34018147 10.1007/s12325-021-01741-7

[32] F. Zieve , N. K. Wenger , O. Ben‐Yehuda , et al., “Safety and Efficacy of Ezetimibe Added to Atorvastatin Versus up Titration of Atorvastatin to 40 mg in Patients ≥65 Years of Age (From the ZETia in the ELDerly [ZETELD] Study),” The American Journal of Cardiology 105, no. 5 (2010): 656–663. 20185012 10.1016/j.amjcard.2009.10.029

[33] N.‐Q. Wu , Y.‐L. Guo , C.‐G. Zhu , et al., “Comparison of Statin Plus Ezetimibe With Double‐Dose Statin on Lipid Profiles and Inflammation Markers,” Lipids in Health and Disease 17 (2018): 265. 30470229 10.1186/s12944-018-0909-z PMC6260646

[34] R. Malmström , M. Settergren , F. Böhm , J. Pernow , and P. Hjemdahl , “No Effect of Lipid Lowering on Platelet Activity in Patients With Coronary Artery Disease and Type 2 Diabetes or Impaired Glucose Tolerance,” Thrombosis and Haemostasis 101, no. 1 (2009): 157–164. 19132203

[35] S. V. Miklishanskaya , T. N. Vlasik , G. I. Kheimets , and V. V. Kukharchuk , “The Possibility of Reducing the Lp‐PLA2 Mass Level Using Simvastatin Monotherapy and Combination Therapy With Ezetimibe,” Coret Vasa 57, no. 4 (2015): e257–e264.

[36] A. E. P. Pesaro , C. V. Serrano Jr. , J. L. Fernandes , et al., “Pleiotropic Effects of Ezetimibe/Simvastatin vs. High Dose Simvastatin,” International Journal of Cardiology 158, no. 3 (2012): 400–404. 21334753 10.1016/j.ijcard.2011.01.062

[37] N. Dagli , M. Yavuzkir , and I. Karaca , “The Effects of High Dose Pravastatin and Low Dose Pravastatin and Ezetimibe Combination Therapy on Lipid, Glucose Metabolism and Inflammation,” Inflammation 30, no. 6 (2007): 230–235. 17687635 10.1007/s10753-007-9041-3

[38] V. Barrios , N. Amabile , F. Paganelli , et al., “Lipid‐Altering Efficacy of Switching From Atorvastatin 10 mg/Day to Ezetimibe/Simvastatin 10/20 mg/Day Compared to Doubling the Dose of Atorvastatin in Hypercholesterolaemic Patients With Atherosclerosis or Coronary Heart Disease,” International Journal of Clinical Practice 59, no. 12 (2005): 1377–1386. 16351668 10.1111/j.1368-5031.2005.00714.x

[39] Y.‐K. Cho , S.‐H. Hur , C.‐D. Han , et al., “Comparison of Ezetimibe/Simvastatin 10/20 mg Versus Atorvastatin 20 mg in Achieving a Target Low Density Lipoprotein‐Cholesterol Goal for Patients With Very High Risk,” Korean Circulation Journal 41, no. 3 (2011): 149–153. 21519514 10.4070/kcj.2011.41.3.149 PMC3079135

[40] S. Fichtlscherer , C. Schmidt‐Lucke , S. Bojunga , et al., “Differential Effects of Short‐Term Lipid Lowering With Ezetimibe and Statins on Endothelial Function in Patients With CAD: Clinical Evidence for ‘Pleiotropic’ functions of Statin Therapy,” European Heart Journal 27, no. 10 (2006): 1182–1190. 16621868 10.1093/eurheartj/ehi881

[41] A. Klassen , A. T. Faccio , C. R. C. Picossi , et al., “Evaluation of Two Highly Effective Lipid‐Lowering Therapies in Subjects With Acute Myocardial Infarction,” Scientific Reports 11, no. 1 (2021): 15973. 34354179 10.1038/s41598-021-95455-z PMC8342504

[42] H. W. O. Roeters van Lennep , A. H. Liem , P. H. J. M. Dunselman , G. M. Dallinga‐Thie , A. H. Zwinderman , and J. Wouter Jukema , “The Efficacy of Statin Monotherapy Uptitration Versus Switching to Ezetimibe/Simvastatin: Results of the EASEGO study,” Current medical research and opinion 24, no. 3 (2008): 685–694. 18226326 10.1185/030079908X273273

[43] M. Oh , H. Kim , E. W. Shin , et al., “Effects of Ezetimibe/Simvastatin 10/10 mg Versus Rosuvastatin 10 mg on Carotid Atherosclerotic Plaque Inflammation,” BMC Cardiovascular Disorders 19, no. 1 (2019): 201. 31426749 10.1186/s12872-019-1184-2 PMC6700958

[44] T. Nakamura , M. Hirano , Y. Kitta , et al., “A Comparison of the Efficacy of Combined Ezetimibe and Statin Therapy With Doubling of Statin Dose in Patients With Remnant Lipoproteinemia on Previous Statin Therapy,” Journal of Cardiology 60, no. 1 (2012): 12–17. 22445441 10.1016/j.jjcc.2012.02.005

[45] J.‐H. Lee , D.‐H. Shin , B.‐K. Kim , et al., “Early Effects of Intensive Lipid‐Lowering Treatment on Plaque Characteristics Assessed by Virtual Histology Intravascular Ultrasound,” Yonsei Medical Journal 57, no. 5 (2016): 1087–1094. 27401638 10.3349/ymj.2016.57.5.1087 PMC4960373

[46] E. Pytel , B. Bukowska , M. Koter‐Michalak , M. Olszewska‐Banaszczyk , P. Gorzelak‐Pabiś , and M. Broncel , “Effect of Intensive Lipid‐Lowering Therapies on Cholinesterase Activity in Patients With Coronary Artery Disease,” Pharmacological Reports 69, no. 1 (2017): 150–155. 27923158 10.1016/j.pharep.2016.09.016

[47] M. Vrablík , I. Šarkanová , K. Breciková , P. Šedová , M. Šatný , and A. Tichopád , “Low LDL‐C goal attainment in Patients at Very High Cardiovascular Risk Due to Lacking Observance of the Guidelines on Dyslipidaemias,” PLoS One 18, no. 5 (2023): e0272883. 37216363 10.1371/journal.pone.0272883 PMC10202298

[48] V. Barrios , X. Pintó , C. Escobar , J. F. Varona , and J. M. Gámez , “Real‐World Attainment of Low‐Density Lipoprotein Cholesterol Goals in Patients at High Risk of Cardiovascular Disease Treated With High‐Intensity Statins: The TERESA Study,” Journal of Clinical Medicine 12, no. 9 (2023): 3187. 37176627 10.3390/jcm12093187 PMC10179558

[49] K. K. Ray , B. Molemans , W. M. Schoonen , et al., “EU‐Wide Cross‐Sectional Observational Study of Lipid‐Modifying Therapy Use in Secondary and Primary Care: The DA VINCI study,” European Journal of Preventive Cardiology 28, no. 11 (2021): 1279–1289. 33580789 10.1093/eurjpc/zwaa047

[50] D. T. Michaeli , J. C. Michaeli , T. Boch , and T. Michaeli , “Cost‐Effectiveness of Icosapent Ethyl, Evolocumab, Alirocumab, Ezetimibe, or Fenofibrate in Combination With Statins Compared to Statin Monotherapy,” Clinical Drug Investigation 42, no. 8 (2022): 643–656. 35819632 10.1007/s40261-022-01173-3 PMC9338124

[51] K. R. Feingold , “Introduction to Lipids and Lipoproteins,” in Endotext, eds. K. R. Feingold , B. Anawalt , and M. R. Blackman (South Dartmouth, MA: MDText.com Inc., 2000).

[52] D. Chinwong , J. Patumanond , S. Chinwong , et al., “Statin Therapy in Patients With Acute Coronary Syndrome: Low‐Density Lipoprotein Cholesterol Goal Attainment and Effect of Statin Potency,” Thermal Clinical Risk Management 11 (2015): 127–136. 10.2147/TCRM.S75608 PMC4315463 25670902

[53] S. Zhan , M. Tang , F. Liu , P. Xia , M. Shu , and X. Wu , “Ezetimibe for the Prevention of Cardiovascular Disease and All‐Cause Mortality Events,” Cochrane Database Systemic Review 11, no. 11 (2018): CD012502. 10.1002/14651858.CD012502.pub2 PMC6516816 30480766

[54] T. Chaiyasothi , S. Nathisuwan , P. Dilokthornsakul , et al., “Effects of Non‐Statin Lipid‐Modifying Agents on Cardiovascular Morbidity and Mortality Among Statin‐Treated Patients: A Systematic Review and Network Meta‐Analysis,” Frontiers in Pharmacology 10 (2019): 547. 31191304 10.3389/fphar.2019.00547 PMC6540916

