# Supplementary material for: Safety and Effectiveness of High‐Intensity Statins Versus Low/Moderate‐Intensity Statins Plus Ezetimibe in Patients With Atherosclerotic Cardiovascular Disease for Reaching LDL‐C Goals: A Systematic Review and Meta‐Analysis
Source: Clin Cardiol. 2024 Aug 12;47(8):e24334. doi: 10.1002/clc.24334 (PMC11319735; doi:10.1002/clc.24334)
Supplement: Supplementary file 1 — Supporting information. [file CLC-47-e24334-s001.docx]

| #1 | ((((((((((((((Artery Disease, Coronary[Title/Abstract]) OR (Artery Diseases, Coronary[Title/Abstract])) OR (Coronary Artery Diseases[Title/Abstract])) OR (Left Main Coronary Artery Disease[Title/Abstract])) OR (Left Main Disease[Title/Abstract])) OR (Left Main Diseases[Title/Abstract])) OR (Left Main Coronary Disease[Title/Abstract])) OR (Coronary Arteriosclerosis[Title/Abstract])) OR (Arterioscleroses, Coronary[Title/Abstract])) OR (Coronary Arterioscleroses[Title/Abstract])) OR (Atherosclerosis, Coronary[Title/Abstract])) OR (Atheroscleroses, Coronary[Title/Abstract])) OR (Coronary Atheroscleroses[Title/Abstract])) OR (Coronary Atherosclerosis[Title/Abstract])) OR (Arteriosclerosis, Coronary[Title/Abstract]) |  |
| --- | --- | --- |
| #2 | ((((((((((((((((((((((((Hydroxymethylglutaryl CoA Reductase Inhibitors[Title/Abstract]) OR (Inhibitors, Hydroxymethylglutaryl-CoA Reductase[Title/Abstract])) OR (Reductase Inhibitors, Hydroxymethylglutaryl-CoA[Title/Abstract])) OR (HMG-CoA Reductase Inhibitor[Title/Abstract])) OR (HMG CoA Reductase Inhibitor[Title/Abstract])) OR (Statin[Title/Abstract])) OR (atorvastatin[Title/Abstract])) OR (rosuvastatin[Title/Abstract])) OR (simvastatin[Title/Abstract])) OR (pitavastatin[Title/Abstract])) OR (pravastatin[Title/Abstract])) OR (lovastatin[Title/Abstract])) OR (HMG-CoA Reductase Inhibitors[Title/Abstract])) OR (HMG CoA Reductase Inhibitors[Title/Abstract])) OR (fluvastatin[Title/Abstract])) OR (Hydroxymethylglutaryl-Coenzyme A Inhibitors[Title/Abstract])) OR (Zocor[Title/Abstract])) OR (Crestor[Title/Abstract])) OR (Hydroxymethylglutaryl-CoA Inhibitors[Title/Abstract])) OR (Livalo[Title/Abstract])) OR (Lescol[Title/Abstract])) OR (Livalo[Title/Abstract])) OR (Altoprev[Title/Abstract])) OR (statins[Title/Abstract])) |  |
| #3 | ((((((((1-(4-fluorophenyl[Title/Abstract])-(3R)-(3-(4-fluorophenyl)-(3S)-hydroxypropyl)-(4S)-(4-hydroxyphenyl)-2-azetidinone)) OR (Ezetimib[Title/Abstract])) OR (Ezetrol[Title/Abstract])) OR (SCH 58235[Title/Abstract])) OR (58235, SCH[Title/Abstract])) OR (SCH-58235[Title/Abstract])) OR (SCH58235[Title/Abstract])) OR (zetia[Title/Abstract]) |  |
| Full search term | #1 AND #2 AND #3 |  |

**Supplementary Table 1. Search terms**

| **Study** | **Double therapy regimen** | **Monotherapy regimen** | **D1** | **D2** | **D3** | **D4** | **D5** | **Overall** |
| --- | --- | --- | --- | --- | --- | --- | --- | --- |
| Lee et al. (2023) | [ezetimibe 10 + rosuvastatin 10] | [rosuvastatin 20] | 🟢 | 🟢 | 🟢 | 🟢 | 🟢 | 🟢 |
| Park et al. (2023) | [ezetimibe 10 + rosuvastatin 10] | [rosuvastatin 20] | 🟢 | 🟢 | 🟢 | 🟢 | 🟢 | 🟢 |
| Qian et al. (2022) | [ezetimibe 10 + atorvastatin 10] [ezetimibe 10 + atorvastatin 20] | [atorvastatin 20] [atorvastatin 40] | 🟢 | 🟢 | 🟢 | 🟢 | 🟢 | 🟢 |
| Klassen et al. (2021) | [simvastatin 40 + ezetimibe10] | [rosuvastatin 20] | 🟢 | 🟢 | 🟢 | 🟢 | 🟢 | 🟢 |
| Tan et al. (2021) | [atorvastatin 10+ezetimibe 10] | [Atorvastatin 40] | 🟢 | 🟢 | 🟢 | 🟢 | 🟢 | 🟢 |
| Oh et al. (2021) | [atorvastatin 10+ezetimibe 10] | [Atorvastatin 40] | 🟢 | 🟢 | 🟢 | 🟢 | 🟢 | 🟢 |
| Oh et al. (2020) | [ezetimibe 10+rosuvastatin 5] | [rosuvastatin 20] | 🟢 | 🟢 | 🟢 | 🟢 | 🟢 | 🟢 |
| Palathingal et al. (2020) | [ezetimibe + low dose atorvastatin] | [high dose atorvastatin] | 🟢 | 🟢 | 🟢 | 🟢 | 🟢 | 🟢 |
| Oh et al. (2019) | [ezetimibe 10 + simvastatin 10] | [rosuvastatin 10] | 🟢 | 🟢 | 🟢 | 🟢 | 🟢 | 🟢 |
| Wu et al. (2018) | [ezetimibe 10 + atorvastatin 20] | [atorvastatin 40] | 🟢 | 🟢 | 🟢 | 🟢 | 🟢 | 🟢 |
| El-Tamalawy et al. (2018) | [ezetimibe 10 + atorvastatin 40] | [atorvastatin 80] | 🟢 | 🟢 | 🟢 | 🟢 | 🟢 | 🟢 |
| Ran et al. (2017) | [ezetimibe 10 + rosuvastatin 10] | [rosuvastatin 20] | 🟢 | 🟢 | 🟢 | 🟢 | 🟢 | 🟢 |
| Japaridze and Sadunishvili (2017) | [atorvastatin 20 + ezetimibe 10] | [atorvastatin 40] | 🟢 | 🟢 | 🟢 | 🟢 | 🟢 | 🟢 |
| Lee et al. (2016) | [ezetimibe 10 + simvastatin 40] | [pravastatin 20] | 🟢 | 🟢 | 🟢 | 🟢 | 🟢 | 🟢 |
| Pytel et al. (2017) | [ezetimibe 10 + atorvastatin 10] | [rosuvastatin 20] [atorvastatin 40] | 🟢 | 🟢 | 🟢 | 🟢 | 🟢 | 🟢 |
| Liu et al. (2017) | N/A | N/A | 🟢 | 🟢 | 🟢 | 🟢 | 🟢 | 🟢 |
| Miklishanskaya et al. (2015) | [ezetimibe 10 + simvastatin 20] | [simvastatin 80] | 🟢 | 🟢 | 🟢 | 🟢 | 🟢 | 🟢 |
| Zieve et al. (2010) | [atorvastatin 10 + ezetimibe 10] | [atorvastatin20] [atorvastatin 40] | 🟢 | 🟢 | 🟢 | 🟢 | 🟢 | 🟢 |
| Yamazaki et al. (2013) | [rosuvastatin 2.5 + ezetimibe 10] | [rosuvastatin 10] | 🟢 | 🟢 | 🟢 | 🟢 | 🟢 | 🟢 |
| Matsue et al. (2013) | [ezetimibe 10 + atorvastatin 10] | [atorvastatin 20] | 🟡 | 🟢 | 🟢 | 🟢 | 🟢 | 🟡 |
| Pesaro et al. (2012) | [ezetimibe 10 + simvastatin20] | [simvastatin 80] | 🟢 | 🟢 | 🟢 | 🟢 | 🟢 | 🟢 |
| Nakamura et al. (2012) | [ezetimibe 10 + any statin] | [same statin with doubled dosage] | 🟢 | 🟢 | 🟢 | 🟢 | 🟢 | 🟢 |
| Cho et al. (2011) | [ezetimibe 10 + simvastatin 20] | [atorvastatin 20] | 🟢 | 🟢 | 🟢 | 🟢 | 🟢 | 🟢 |
| Foody et al. (2010) | [ezetimibe 10 + simvastatin 20] [ezetimibe 10 + simvastatin 40] | [atorvastatin 10] [atorvastatin 20] [atorvastatin 40] | 🟢 | 🟢 | 🟢 | 🟢 | 🟢 | 🟢 |
| Pandey et al. (2011) | [ezetimibe 10 + any statin] | [same statin with doubled dosage] | 🟢 | 🟡 | 🟢 | 🟢 | 🟢 | 🟡 |
| Ostad et al. (2009) | [atorvastatin 10+ ezetimibe 10] | [atorvastatin 80] | 🟢 | 🟢 | 🟡 | 🟢 | 🟢 | 🟡 |
| Malmström et al. (2009) | [ezetimibe 10 + Simvastatin 10] | [simvastatin 80] | 🟢 | 🟢 | 🟢 | 🟢 | 🟢 | 🟢 |
| Roeters van Lennep et al. (2008) | [ezetimibe 10 + simvastatin 20] | [simvastatin 40] [atorvastatin 20] | 🟢 | 🟢 | 🟢 | 🟢 | 🟢 | 🟢 |
| Piorkowski et al. (2007) | [ezetimibe 10 + atorvastatin 10] | [atorvastatin 40] | 🟢 | 🟢 | 🟢 | 🟢 | 🟢 | 🟢 |
| Dagli et al. (2007) | [ezetimibe 10 + pravastatin 10] | [pravastatin 40] | 🟢 | 🟢 | 🟢 | 🟢 | 🟢 | 🟢 |
| Fichtlscherer et al. (2006) | [ezetimibe 10 + simvastatin 20] | [atorvastatin 40] | 🟡 | 🟢 | 🟢 | 🟢 | 🟢 | 🟡 |
| Barrios et al. (2005) | [ezetimibe 10 + simvastatin 20] | [atorvastatin 20] | 🟢 | 🟢 | 🟢 | 🟢 | 🟢 | 🟢 |

**Supplementary Table 2. Risk of bias assessment of the included RCTs conducted using Rob2. Green color stands for low risk of bias, yellow color for some concerns and red color for high risk of bias.**

Barrios, V., Amabile, N., Paganelli, F., Chen, J. W., Allen, C., Johnson-Levonas, A. O., Massaad, R., & Vandormael, K. (2005). Lipid-altering efficacy of switching from atorvastatin 10 mg/day to ezetimibe/simvastatin 10/20 mg/day compared to doubling the dose of atorvastatin in hypercholesterolaemic patients with atherosclerosis or coronary heart disease. *Int J Clin Pract*, *59*(12), 1377-1386. <https://doi.org/doi:10.1111/j.1368-5031.2005.00714.x>

Cho, Y. K., Hur, S. H., Han, C. D., Park, H. S., Yoon, H. J., Kim, H., Nam, C. W., Kim, Y. N., Kim, K. B., Park, N. H., & Park, H. J. (2011). Comparison of Ezetimibe/Simvastatin 10/20 mg Versus Atorvastatin 20 mg in Achieving a Target Low Density Lipoprotein-Cholesterol Goal for Patients With Very High Risk [Article]. *Korean Circulation Journal*, *41*(3), 149-153. <https://doi.org/10.4070/kcj.2011.41.3.149>

Dagli, N., Yavuzkir, M., & Karaca, I. (2007). The effects of high dose pravastatin and low dose pravastatin and ezetimibe combination therapy on lipid, glucose metabolism and inflammation. *Inflammation*, *30*(6), 230-235. <https://doi.org/doi:10.1007/s10753-007-9041-3>

El-Tamalawy, M. M., Ibrahim, O. M., Hassan, T. M., & El-Barbari, A. A. (2018). Effect of Combination Therapy of Ezetimibe and Atorvastatin on Remnant Lipoprotein Versus Double Atorvastatin Dose in Egyptian Diabetic Patients. *J Clin Pharmacol*, *58*(1), 34-41. <https://doi.org/doi:10.1002/jcph.976>

Fichtlscherer, S., Schmidt-Lucke, C., Bojunga, S., Rössig, L., Heeschen, C., Dimmeler, S., & Zeiher, A. M. (2006). Differential effects of short-term lipid lowering with ezetimibe and statins on endothelial function in patients with CAD: clinical evidence for 'pleiotropic' functions of statin therapy. *Eur Heart J*, *27*(10), 1182-1190. <https://doi.org/doi:10.1093/eurheartj/ehi881>

Foody, J. M., Brown, W. V., Zieve, F., Adewale, A. J., Flaim, D., Lowe, R. S., Jones-Burton, C., & Tershakovec, A. M. (2010). Safety and efficacy of ezetimibe/simvastatin combination versus atorvastatin alone in adults ≥65 years of age with hypercholesterolemia and with or at moderately high/high risk for coronary heart disease (the VYTELD study). *Am J Cardiol*, *106*(9), 1255-1263. <https://doi.org/10.1016/j.amjcard.2010.06.051>

Japaridze, L., & Sadunishvili, M. (2017). The short-term effect of atorvastatin plus ezetimibe therapy versus atorvastatin monotherapy on clinical outcome in acute coronary syndrome patients by gender. *Kardiol Pol*, *75*(8), 770-778. <https://doi.org/10.5603/KP.a2017.0074>

Klassen, A., Faccio, A. T., Picossi, C. R. C., Derogis, P., Dos Santos Ferreira, C. E., Lopes, A. S., Sussulini, A., Cruz, E. C. S., Bastos, R. T., Fontoura, S. C., Neto, A. M. F., Tavares, M. F. M., Izar, M. C., & Fonseca, F. A. H. (2021). Evaluation of two highly effective lipid-lowering therapies in subjects with acute myocardial infarction. *Sci Rep*, *11*(1), 15973. <https://doi.org/10.1038/s41598-021-95455-z>

Lee, J. H., Shin, D. H., Kim, B. K., Ko, Y. G., Choi, D., Hong, M. K., & Jang, Y. (2016). Early effects of intensive lipid-lowering treatment on plaque characteristics assessed by virtual histology intravascular ultrasound [Conference Abstract]. *European Heart Journal*, *37*, 487. <https://doi.org/10.1093/eurheartj/ehw432>

Lee, S. J., Cha, J. J., Choi, W. G., Lee, W. S., Jeong, J. O., Choi, S., Cho, Y. H., Park, W., Yoon, C. H., Lee, Y. J., Hong, S. J., Ahn, C. M., Kim, B. K., Ko, Y. G., Choi, D., Hong, M. K., Jang, Y., Hong, S. J., & Kim, J. S. (2023). Moderate-Intensity Statin With Ezetimibe Combination Therapy vs High-Intensity Statin Monotherapy in Patients at Very High Risk of Atherosclerotic Cardiovascular Disease: A Post Hoc Analysis From the RACING Randomized Clinical Trial. *JAMA Cardiol*, *8*(9), 853-858. <https://doi.org/10.1001/jamacardio.2023.2222>

Liu, Z., Hao, H., Yin, C., Chu, Y., Li, J., & Xu, D. (2017). Therapeutic effects of atorvastatin and ezetimibe compared with double-dose atorvastatin in very elderly patients with acute coronary syndrome. *Oncotarget*, *8*(25), 41582-41589. <https://doi.org/10.18632/oncotarget.15078>

Malmström, R. E., Settergren, M., Böhm, F., Pernow, J., & Hjemdahl, P. (2009). No effect of lipid lowering on platelet activity in patients with coronary artery disease and type 2 diabetes or impaired glucose tolerance. *Thromb Haemost*, *101*(1), 157-164.

Matsue, Y., Matsumura, A., Suzuki, M., Hashimoto, Y., & Yoshida, M. (2013). Differences in action of atorvastatin and ezetimibe in lowering low-density lipoprotein cholesterol and effect on endothelial function: randomized controlled trial. *Circ J*, *77*(7), 1791-1798. <https://doi.org/10.1253/circj.cj-13-0033>

Miklishanskaya, S. V., Vlasik, T. N., Kheimets, G. I., & Kukharchuk, V. V. (2015). The possibility of reducing the Lp-PLA2 mass level using simvastatin monotherapy and combination therapy with ezetimibe. *Cor Et Vasa*, *57*(4), e257-e264. <https://doi.org/https://doi.org/10.1016/j.crvasa.2015.03.012>

Nakamura, T., Hirano, M., Kitta, Y., Fujioka, D., Saito, Y., Kawabata, K., Obata, J., Watanabe, Y., Watanabe, K., & Kugiyama, K. (2012). A comparison of the efficacy of combined ezetimibe and statin therapy with doubling of statin dose in patients with remnant lipoproteinemia on previous statin therapy [Article]. *Journal of Cardiology*, *60*(1-2), 12-17. <https://doi.org/10.1016/j.jjcc.2012.02.005>

Oh, M., Kim, H., Shin, E. W., Sung, C., Kim, D. H., Moon, D. H., Kim, N., Eo, J. S., Kim, J. W., & Lee, C. W. (2020). Comparison of High-Dose Rosuvastatin Versus Low-Dose Rosuvastatin Plus Ezetimibe on Carotid Atherosclerotic Plaque Inflammation in Patients with Acute Coronary Syndrome. *J Cardiovasc Transl Res*, *13*(6), 900-907. <https://doi.org/doi:10.1007/s12265-020-10009-4>

Oh, M., Kim, H., Shin, E. W., Sung, C., Kim, D. H., Moon, D. H., & Lee, C. W. (2019). Effects of ezetimibe/simvastatin 10/10 mg versus Rosuvastatin 10 mg on carotid atherosclerotic plaque inflammation. *BMC Cardiovasc Disord*, *19*(1), 201. <https://doi.org/10.1186/s12872-019-1184-2>

Oh, P. C., Jang, A. Y., Ha, K., Kim, M., Moon, J., Suh, S. Y., Lee, K., Han, S. H., & Kang, W. C. (2021). Effect of Atorvastatin (10 mg) and Ezetimibe (10 mg) Combination Compared to Atorvastatin (40 mg) Alone on Coronary Atherosclerosis. *Am J Cardiol*, *154*, 22-28. <https://doi.org/10.1016/j.amjcard.2021.05.039>

Ostad, M. A., Eggeling, S., Tschentscher, P., Schwedhelm, E., Böger, R., Wenzel, P., Meinertz, T., Munzel, T., & Warnholtz, A. (2009). Flow-mediated dilation in patients with coronary artery disease is enhanced by high dose atorvastatin compared to combined low dose atorvastatin and ezetimibe: results of the CEZAR study. *Atherosclerosis*, *205*(1), 227-232. <https://doi.org/doi:10.1016/j.atherosclerosis.2008.11.032>

Palathingal, J. T., Vijayan, D., Drisya Rajan, C., Stanly, I., Jamshad, T. P., & Linu, M. P. (2020). A randomised controlled study of high dose statin versus statin plus ezetimibe therapy in patients with acute coronary syndrome. *International Journal of Biomedical Science*, *16*(4), 52-67. <https://doi.org/doi>:

Pandey, A. S., Bissonnette, S., Boukas, S., Rampakakis, E., & Sampalis, J. S. (2011). Effectiveness and tolerability of ezetimibe co-administered with statins versus statin dose-doubling in high-risk patients with persistent hyperlipidemia: The EZE(STAT)2 trial [Article]. *Archives of Medical Science*, *7*(5), 767-775. <https://doi.org/10.5114/aoms.2011.25550>

Park, J. I., Lee, S. J., Hong, B. K., Cho, Y. H., Shin, W. Y., Lim, S. W., Kang, W. C., Park, Y., Lee, S. Y., Lee, Y. J., Hong, S. J., Ahn, C. M., Kim, B. K., Ko, Y. G., Choi, D., Hong, M. K., Jang, Y., & Kim, J. S. (2023). Efficacy and safety of moderate-intensity statin with ezetimibe combination therapy in patients after percutaneous coronary intervention: a post-hoc analysis of the RACING trial. *EClinicalMedicine*, *58*, 101933. <https://doi.org/10.1016/j.eclinm.2023.101933>

Pesaro, A. E., Serrano, C. V., Jr., Fernandes, J. L., Cavalcanti, A. B., Campos, A. H., Martins, H. S., Maranhão, R. C., de Lemos, J. A., Souza, H. P., & Nicolau, J. C. (2012). Pleiotropic effects of ezetimibe/simvastatin vs. high dose simvastatin. *Int J Cardiol*, *158*(3), 400-404. <https://doi.org/10.1016/j.ijcard.2011.01.062>

Piorkowski, M., Fischer, S., Stellbaum, C., Jaster, M., Martus, P., Morguet, A. J., Schultheiss, H. P., & Rauch, U. (2007). Treatment with ezetimibe plus low-dose atorvastatin compared with higher-dose atorvastatin alone - Is sufficient cholesterol-lowering enough to inhibit platelets? [Article]. *Journal of the American College of Cardiology*, *49*(10), 1035-1042. <https://doi.org/10.1016/j.jacc.2006.10.064>

Pytel, E., Bukowska, B., Koter-Michalak, M., Olszewska-Banaszczyk, M., Gorzelak-Pabiś, P., & Broncel, M. (2017). Effect of intensive lipid-lowering therapies on cholinesterase activity in patients with coronary artery disease. *Pharmacol Rep*, *69*(1), 150-155. <https://doi.org/doi:10.1016/j.pharep.2016.09.016>

Qian, J., Li, Z., Zhang, X., Chen, J., Ding, C., Yang, P., Liu, Y., Shi, M., Ren, X., & Ge, J. (2022). Efficacy and Tolerability of Ezetimibe/Atorvastatin Fixed-dose Combination Versus Atorvastatin Monotherapy in Hypercholesterolemia: A Phase III, Randomized, Active-controlled Study in Chinese Patients. *Clin Ther*, *44*(10), 1282-1296. <https://doi.org/10.1016/j.clinthera.2022.08.013>

Ran, D., Nie, H. J., Gao, Y. L., Deng, S. B., Du, J. L., Liu, Y. J., Jing, X. D., & She, Q. (2017). A randomized, controlled comparison of different intensive lipid-lowering therapies in Chinese patients with non-ST-elevation acute coronary syndrome (NSTE-ACS): Ezetimibe and rosuvastatin versus high-dose rosuvastatin. *Int J Cardiol*, *235*, 49-55. <https://doi.org/doi:10.1016/j.ijcard.2017.02.099>

Roeters van Lennep, H. W., Liem, A. H., Dunselman, P. H., Dallinga-Thie, G. M., Zwinderman, A. H., & Jukema, J. W. (2008). The efficacy of statin monotherapy uptitration versus switching to ezetimibe/simvastatin: results of the EASEGO study. *Curr Med Res Opin*, *24*(3), 685-694. <https://doi.org/10.1185/030079908x273273>

Tan, H., Liu, L., Zheng, Q., Zhang, D., Liu, Q., Cui, D., Gao, L., Wang, Z., Wang, W. L., & Liu, J. (2021). Effects of Combined Lipid-Lowering Therapy on Low-Density Lipoprotein Cholesterol Variability and Cardiovascular Adverse Events in Patients with Acute Coronary Syndrome. *Adv Ther*, *38*(6), 3389-3398. <https://doi.org/10.1007/s12325-021-01741-7>

Wu, N. Q., Guo, Y. L., Zhu, C. G., Gao, Y., Zhao, X., Sun, D., Sun, J., Xu, R. X., Liu, G., Dong, Q., & Li, J. J. (2018). Comparison of statin plus ezetimibe with double-dose statin on lipid profiles and inflammation markers. *Lipids Health Dis*, *17*(1), 265. <https://doi.org/10.1186/s12944-018-0909-z>

Yamazaki, D., Ishida, M., Watanabe, H., Nobori, K., Oguma, Y., Terata, Y., Koyama, T., Iino, K., Kosaka, T., & Ito, H. (2013). Comparison of anti-inflammatory effects and high-density lipoprotein cholesterol levels between therapy with quadruple-dose rosuvastatin and rosuvastatin combined with ezetimibe. *Lipids Health Dis*, *12*, 9. <https://doi.org/10.1186/1476-511x-12-9>

Zieve, F., Wenger, N. K., Ben-Yehuda, O., Constance, C., Bird, S., Lee, R., Hanson, M. E., Jones-Burton, C., & Tershakovec, A. M. (2010). Safety and efficacy of ezetimibe added to atorvastatin versus up titration of atorvastatin to 40 mg in Patients > or = 65 years of age (from the ZETia in the ELDerly [ZETELD] study). *Am J Cardiol*, *105*(5), 656-663. <https://doi.org/10.1016/j.amjcard.2009.10.029>

| Klassen, Faccio et al. (2021) | Qian, Li et al. (2022) | Park, Lee et al. (2023) | Lee, Seung-Jun et al.* (2023) | First author (publication year) | Study characteristic |
| --- | --- | --- | --- | --- | --- |
| Brazil | China | South Korea | South Korea | Country |  |
| 1 | 3 | 36 | 36 | Treatment duration (months) |  |
| 20 | 454 | 2497 | 1)2269  2)1511 | Total sample size |  |
| ACS | ASCVD | ASCVD | ASCVD | Background disease |  |
| 10 | 225 | 1258 | 1)1137  2)757 | No. | Intervention group |
| 53(48-62) | EZ10/A10:62.4±7.4 EZ10/AS20: 59.4±9.3 | 63.8±9.5 | 1)63.5±9.3  2)63.6±9.9 | Mean age ±  SD, range |  |
| 80 | EZ10/A10: 51.1. EZ10/A20:75.2 | 79 | 1)70.7  2)81.4 | Gender (males%) |  |
| 60 | N/A | 41.7 | 1)32.2  2)44.1 | No.(%) DM |  |
| 70 | EZ10/A10:65.9. EZ10/A20:66.4 | 69 | 1)59.5  2)75.2 | No.(%) HTN |  |
| [Simvastatin 40 + Ezetimibe 10] | [Ezetimibe 10 + Atorvastatin 10] [Ezetimibe 10 + Atorvastatin 20] | [Ezetimibe 10 + Rosuvastatin 10] | [Ezetimibe 10 + Rosuvastatin 10] | Medication |  |
| 10 | 229 | 1239 | 1)1132  2)754 | No. | Control group |
| 62(59-64) | A20:62.4±8.5 A40:60.6±10.3 | 64.6±9.6 | 1)63.9±9.2  2)64.3±10.3 | Mean age ±  SD, range |  |
| 70 | A20:57.3  A40:67.1 | 78.4 | 1)71.2  2)79.6 | Gender (males%) |  |
| 40 | N/A | 41.1 | 1)32.7  2)43.4 | No.(%) DM |  |
| 70 | A20:70.8  A40:66.4 | 70.2 | 1)61.8  2)76.1 | No.(%) HTN |  |
| [Rosuvastatin 20] | [Atorvastatin 20] [Atorvastatin 40] | [Rosuvastatin 20] | [Rosuvastatin 20] | Medication |  |
| Palathingal, Vijayan et al. (2020) | Oh, Kim et al. (2020) | Oh, Jang et al. (2021) | Tan, Liu et al. (2021) | First author (publication year) | Study characteristic |
| India | South Korea | Korea | China | Country |  |
| 6 | 6 | 12 | 24 | Treatment duration (months) |  |
| 68 | 50 | 37 | 183 | Total sample size |  |
| ACS | ACS | ACS | ACS | Background disease |  |
| 30 | 25 | 18 | 91 | No. | Intervention group |
| 64.37±10.3 | 59.6±9.9 | 56.3±7.1 | 49.83±9.87 | Mean age ±  SD, range |  |
| N/A | 84 | 72 | 56.04 | Gender (males%) |  |
| N/A | 16.7 | 33 | 71.43 | No.(%) DM |  |
| N/A | 45.8 | 56 | 23.08 | No.(%) HTN |  |
| [Ezetimibe + Low dose Atorvastatin] | [Ezetimibe 10 + Rosuvastatin 5] | [Atorvastatin 10 + Ezetimibe 10] | [Atorvastatin 10 + Ezetimibe 10] | Medication |  |
| 38 | 25 | 19 | 92 | No. | Control group |
| 64.73±9.73 | 59.2±9.7 | 56.7±8.4 | 48.08±9.01 | Mean age ±  SD, range |  |
| N/A | 92 | 79 | 54.35 | Gender (males%) |  |
| N/A | 20 | 26 | 68.48 | No.(%) DM |  |
| N/A | 28 | 53 | 18.48 | No.(%) HTN |  |
| [High dose atorvastatin] | [Rosuvastatin 20] | [Atorvastatin 40] | [Atorvastatin 40] | Medication |  |
| Ran, Nie et al. (2017) | El-Tamalawy, Ibrahim et al. (2018) | Wu, Guo et al. (2018) | Oh, Kim et al. (2019) | First author (publication year) | Study characteristic |
| China | Egypt | China | South Korea | Country |  |
| 3 | 3 | 3 | 6 | Treatment duration (months) |  |
| 83 | 65 | 98 | 50 | Total sample size |  |
| ACS | ASCVD | ASCVD | ACS, Hypertension | Background disease |  |
| 42 | 33 | 48 | 25 | No. | Intervention group |
| 60.4±8.2 | 61±7.1 | 56±11 | 62.5±7.4 | Mean age ±  SD, range |  |
| 76.2 | 55 | 72.9 | 88 | Gender (males%) |  |
| 26.2 | N/A | N/A | 8 | No.(%) DM |  |
| 50 | N/A | N/A | 68 | No.(%) HTN |  |
| [Ezetimibe 10 + Rosuvastatin 10] | [Ezetimibe 10 + Atorvastatin 40] | [Ezetimibe 10 + Atorvastatin 20] | [Ezetimibe 10 + Simvastatin 10] | Medication |  |
| 41 | 32 | 50 | 25 | No. | Control group |
| 60.5±10 | 62±6.9 | 57±8 | 59.2±8.8 | Mean age ±  SD, range |  |
| 73.2 | 70 | 72 | 84 | Gender (males%) |  |
| 26.8 | N/A | N/A | 12 | No.(%) DM |  |
| 48.8 | N/A | N/A | 40 | No.(%) HTN |  |
| [Rosuvastatin 20] | [Atorvastatin 80] | [Atorvastatin 40] | [Rosuvastatin 10] | Medication |  |
| Liu, Hao et al. (2017) | Pytel, Bukowska et al. (2017) | Lee, Shin et al. (2016) | Japaridze and Sadunishvili (2017) | First author (publication year) | Study characteristic |
| China | Poland | Korea | Georgia | Country |  |
| 12 | 6 | 3 | 4 | Treatment duration (months) |  |
| 230 | 61 | 70 | 292 | Total sample size |  |
| ACS | N/A | ACS | ACS | Background disease |  |
| 114 | 20 | 34 | 146 | No. | Intervention group |
| 84.2±2.9 | N/A | 60.9±10.9 | 62.21±11.36 | Mean age ±  SD, range |  |
| 52.6 | N/A | 79.4 | 54.1 | Gender (males%) |  |
| 40.4 | N/A | 32.4 | 4.8 | No.(%) DM |  |
| 71.1 | N/A | 50 | N/A | No.(%) HTN |  |
| [Ezetimibe 10 + Atorvastatin 10] | [Ezetimibe 10 + Atorvastatin 10] | [Ezetimibe 10 + Simvastatin 40] | [Atorvastatin 20 + Ezetimibe 10] | Medication |  |
| 116 | 41 | 36 | 146 | No. | Control group |
| 84±1.8 | N/A | 59.3±10.7 | 62.62±11.03 | Mean age ±  SD, range |  |
| 50.9 | N/A | 75 | 53.1 | Gender (males%) |  |
| 36.2 | N/A | 25 | 1.4 | No.(%) DM |  |
| 69 | N/A | 58.3 | N/A | No.(%) HTN |  |
| [Atorvastatin 20] | [Rosuvastatin 20] [Atorvastatin 40] | [Pravastatin 20] | [Atorvastatin 40] | Medication |  |
| Matsue, Matsumura et al. (2013) | Yamazaki, Ishida et al. (2013) | Zieve, Wenger et al. (2010) | Miklishanskaya, Vlasik et al. (2015) | First author (publication year) | Study characteristic |
| Japan | Japan | United States, Russia, Ukraine, Canada, Romania, Poland | Russia | Country |  |
| 3 | 3 | 3 | 6 | Treatment duration (months) |  |
| 243 | 46 | 1053 | 100 | Total sample size |  |
| ASCVD | ASCVD | ASCVD | ASCVD | Background disease |  |
| 115 | 22 | 526 | 50 | No. | Intervention group |
| 69.2±9.3 | 70.1±9.6 | 71±5 | 62(56-69) | Mean age ±  SD, range |  |
| 72.2 | 63.6 | 47 | 74 | Gender (males%) |  |
| 36.5 | 36.4 | 21 | 14 | No.(%) DM |  |
| 78.3 | 77 | N/A | 94 | No.(%) HTN |  |
| [Ezetimibe 10 + Atorvastatin 10] | [Rosuvastatin 2.5 + Ezetimibe 10] | [Atorvastatin 10 + Ezetimibe 10] | [Ezetimibe 10 + Simvastatin 20] | Medication |  |
| 128 | 24 | 527 | 50 | No. | Control group |
| 70.3±9.9 | 71.8±8.2 | 71±5 | 61(54-65) | Mean age ±  SD, range |  |
| 75 | 62.5 | 46 | 78 | Gender (males%) |  |
| 40.6 | 41.7 | 21 | 14 | No.(%) DM |  |
| 81.2 | 79 | N/A | 90 | No.(%) HTN |  |
| [Atorvastatin 20] | [Rosuvastatin 10] | [Atorvastatin 20] [Atorvastatin 40] | [Simvastatin 80] | Medication |  |
| Foody, Brown et al. (2010) | Cho, Hur et al. (2011) | Nakamura, Hirano et al. (2012) | Pesaro, Serrano et al. (2012) | First author (publication year) | Study characteristic |
| Massachusetts, Georgia, Virginia, Pennsylvania | Korea | Japan | Brazil | Country |  |
| 3 | 1.5 | 6 | 1.5 | Treatment duration (months) |  |
| 1289 | 74 | 57 | 78 | Total sample size |  |
| ASCVD | ASCVD | ASCVD | ASCVD | Background disease |  |
| 516 | 36 | 29 | 40 | No. | Intervention group |
| EZ10/S20: 71.8±55 EZ10/S40; 72.2±5.6 | 60.5±9.5 | 61±10 | 64.5±9 | Mean age ±  SD, range |  |
| EZ10/S20:44 EZ10/S40:40 | 66.7 | 75.9 | 68 | Gender (males%) |  |
| EZ10/S20:15 EZ10/S40: 15 | 33.3 | 34.5 | 40 | No.(%) DM |  |
| N/A | 50 | 62.1 | 90 | No.(%) HTN |  |
| [Ezetimibe 10 + Simvastatin 20] [Ezetimibe 10 + Simvastatin 40] | [Ezetimibe 10 + Simvastatin 20] | [Ezetimibe 10 + Any statin] | [Ezetimibe 10 + Simvastatin 20]] | Medication |  |
| 773 | 38 | 28 | 38 | No. | Control group |
| A10:72.1±5.7 A20:71.7±5.2 A40:72.1±5.1 | 62.6±9.7 | 64±9.2 | 61.7±10 | Mean age ±  SD, range |  |
| A10:33 A20:32 A40:36 | 57.9 | 82.1 | 55 | Gender (males%) |  |
| A10:10 A20:16 A40:12 | 34.2 | 37 | 52 | No.(%) DM |  |
| N/A | 55.3 | 60.7 | 68 | No.(%) HTN |  |
| [Atorvastatin 10] [Atorvastatin 20] [Atorvastatin 40] | [Atorvastatin 20] | [Same statin with doubled dosage] | [Simvastatin 80] | Medication |  |
| Roeters van Lennep, Liem et al. (2008) | Malmström, Settergren et al. (2009) | Ostad, Eggeling et al. (2009) | Pandey, Bissonnette et al. (2011) | First author (publication year) | Study characteristic |
| Netherlands | Sweden | Germany | Canada | Country |  |
| 3 | 1.5 | 2 | 1.5 | Treatment duration (months) |  |
| 367 | 32 | 49 | 936 | Total sample size |  |
| ASCVD | ASCVD, diabetes | ASCVD | ASCVD, diabetes | Background disease |  |
| 178 | 15 | 25 | 620 | No. | Intervention group |
| 64±10 | 74(66-77) | 64±10 | 63±11.3 | Mean age ±  SD, range |  |
| 75 | 60 | 76 | 63.2 | Gender (males%) |  |
| 11 | 100 | 16 | 20.8 | No.(%) DM |  |
| 39 | N/A | 68 | 55.6 | No.(%) HTN |  |
| [Ezetimibe 10 + Simvastatin 20] | [Ezetimibe 10 + Simvastatin 10] | [Atorvastatin 10 + Ezetimibe 10] | [Ezetimibe 10 + Any statin] | Medication |  |
| 189 | 17 | 24 | 316 | No. | Control group |
| 65±10 | 70(67-74) | 66±9 | 63±11.4 | Mean age ±  SD, range |  |
| 76 | 76.47 | 79 | 60.4 | Gender (males%) |  |
| 13 | 82.35 | 25 | 26.3 | No.(%) DM |  |
| 47 | N/A | 88 | 56.6 | No.(%) HTN |  |
| [Simvastatin 40] [Atorvastatin 20] | [Simvastatin 80] | [Atorvastatin 80] | [Same statin with doubled dosage] | Medication |  |
| Barrios, Amabile et al. (2005) | Fichtlscherer, Schmidt-Lucke et al. (2006) | Dagli, Yavuzkir et al. (2007) | Piorkowski, Fischer et al. (2007) | First author (publication year) | Study characteristic |
| Spain, France, Taipei, USA, Belgium | Germany | Turkey | Germany | Country |  |
| 1.5 | 1 | 6 | 1 | Treatment duration (months) |  |
| 435 | 30 | 100 | 51 | Total sample size |  |
| ASCVD | ASCVD | ASCVD | ASCVD | Background disease |  |
| 221 | 15 | 50 | 26 | No. | Intervention group |
| 63.5±9.6 | 55.2±6.6 | 53.2±12.2 | 62±2.1 | Mean age ±  SD, range |  |
| 63.8 | 67 | 46 | 76.92 | Gender (males%) |  |
| 26.7 | 13 | N/A | 15.38 | No.(%) DM |  |
| 63.8 | 33 | N/A | 100 | No.(%) HTN |  |
| [Ezetimibe 10 + Simvastatin 20] | [Ezetimibe 10 + Simvastatin 20] | [Ezetimibe 10 + Pravastatin 10] | [Ezetimibe 10 + Atorvastatin 10] | Medication |  |
| 214 | 15 | 50 | 25 | No. | Control group |
| 63.4±10.2 | 56.5±3.9 | 57.1±11.1 | 61.4±1.8 | Mean age ±  SD, range |  |
| 59.8 | 80 | 52 | 88 | Gender (males%) |  |
| 24.8 | 0 | N/A | 28 | No.(%) DM |  |
| 54.2 | 53 | N/A | 100 | No.(%) HTN |  |
| [Atorvastatin 20] | [Atorvastatin 40] | [Pravastatin 40] | [Atorvastatin 40] | Medication |  |

**Supplementary Table 3. Study characteristics**

(ACS=acute coronary syndrome, ASCVD=atherosclerotic cardiovascular disease, DM=diabetes mellitus, HTN=hypertension) *This study included two populations of 1) documented atherosclerotic cardiovascular disease history without very high risk for ASCVD and 2) documented atherosclerotic cardiovascular disease history with very high risk for ASCVD

| **Subgroups** | **Number of trials** | **MD (95% CI)** | **I^2^ (%)** | **P-value for heterogeneity** |
| --- | --- | --- | --- | --- |
| *LDL-C* | | | | |
| Drug type  Rosuvastatin  Atorvastatin  Multiple statins  Simvastatin  Pravastatin | 5  9  6  1  1 | -8.2 [-12.4; -4.1]  -10.1 [-15.1; -5.1]  -0.3 [-10.7; 10.2]  11.6 [-4.7; 27.9]  -16.5 [-26.9; -6.1] | 73  71  88 | <0.01 |
| Duration of follow-up  <12 months  >12 months | 17  4 | -6.4 [-11.8; -0.9]  -8.5 [-10.1; -7.0] | 87  48 | <0.01 |
| *HDL-C* | | | | |
| Drug type  Rosuvastatin  Atorvastatin  Multiple statins  Simvastatin  Pravastatin | 3  7  6  1  1 | 0.8 [-1.5; 3.1]  7.3 [-3.6; 18.1]  0.9 [-2.4; 4.2]  0 [-11.0; 11.0]  -2 [-5.7; 1.7] | 0  100  82 | 0 |
| Duration of follow-up  <12 months  >12 months | 15  3 | 1.4 [-0.4; 3.3]  10.8 [-16.2; 37.9] | 69  100 | 0 |
| *TG* | | | | |
| Drug type  Rosuvastatin  Atorvastatin  Multiple statins  Simvastatin  Pravastatin | 3  6  6  1  1 | -1.1 [ -12.3; 10.0]  -9.9 [-29.4; 9.5]  5.4 [-13.4; 24.2]  0 [-30.7; 30.7]  -36.3 [-59.0; -13.6] | 0  83  75 | <0.01 |
| Duration of follow-up  <12 months  >12 months | 15  1 | -4.4 [-15.9; 7.1]  -8.8 [-29.4; 11.7] | 79 | <0.01 |
| *TC* | | | | |
| Drug type  Rosuvastatin  Atorvastatin  Multiple statins  Simvastatin  Pravastatin | 3  7  6  1  1 | -2.5 [-9.9; 4.8]  -1.8 [-31.3; 27.7]  5.1 [-10.4; 20.5]  11.6 [-9.8; 33.0]  -23.4 [-37.5; -9.3] | 5  99  87 | <0.01 |
| Duration of follow-up  <12 months  >12 months | 15  2 | 0.3 [-10.2; 10.9]  -6.1 [-105.9; 93.8] | 90  99 | <0.01 |
| *hs-CRP* | | | | |
| Drug type  Rosuvastatin  Atorvastatin  Multiple statins  Simvastatin  Pravastatin | 3  5  4  1  1 | -0.0 [-0.0; -0.0]  -0.0 [-0.0; 0.0]  -0.0 [-0.0; 0.0]  0.8 [-1.6; 3.2]  -0.3 [-1.0; 0.3] | 0  33  0 | 0.13 |
| Duration of follow-up  <12 months  >12 months | 12  2 | -0.0 [-0.00; 0.0]  -0.0 [-0.0; -0.0] | 1  0 | 0.13 |

**Supplementary Table 4. Subgroup analyses of different used statins and treatment periods** (CI=confidence interval, HDL-C= high density lipoprotein cholesterol, HR=hazard ratio, hs-CRP=high sensitivity C-reactive protein, LDL-C=low density lipoprotein cholesterol, MD=mean difference, TC=total cholesterol, TG=triglycerides)

| **Subgroups** | **Number of trials** | **RR (95% CI)** | **I^2^ (%)** | **P-value for heterogeneity** |
| --- | --- | --- | --- | --- |
| *Discontinuation of treatment* | | | | |
| Drug type  Rosuvastatin  Atorvastatin  Multiple statins  Pravastatin | 4  6  3  1 | 0.57 [0.47; 0.69]  0.64 [0.36; 1.15]  1.47 [0.73; 2.94]  - | 0  0  0 | 0.61 |
| Duration of follow-up  <12 months  >12 months | 11  3 | 0.87 [0.56; 1.35]  0.57 [0.47; 0.70] | 0  0 | 0.61 |

**Supplementary Table 5. Subgroup analyses of different used statins and treatment periods (CI=confidence interval, RR=risk ratio)**

Supplementary Figure 1. (A) Mean difference in LDL-C funnel plot (B) Mean difference in HDL-C funnel plot (C) Mean difference in TG funnel plot (D) Mean difference in TC funnel plot (E) Mean difference in hs-CRP funnel plot

Supplementary Figure 2. (A) Mean difference in LDL-C for treatment duration subgroup forest plot (B) Mean difference in LDL-C for drug type subgroup forest plot

Supplementary Figure 3. (A) Mean difference in HDL-C for treatment duration subgroup forest plot (B) Mean difference in HDL-C for drug type subgroup forest plot

Supplementary Figure 4. (A) Mean difference in TG for treatment duration subgroup forest plot (B) Mean difference in TG for drug type subgroup forest plot

Supplementary Figure 5. (A) Mean difference in TC for treatment duration subgroup forest plot (B) Mean difference in TC for drug type subgroup forest plot

Supplementary Figure 6. (A) Mean difference in hs-CRP for treatment duration subgroup forest plot (B) Mean difference in hs-CRP for drug type subgroup forest plot

Supplementary Figure 7. (A) Therapy discontinuation for treatment duration subgroup forest plot (B) Therapy discontinuation for drug type subgroup forest plot

Supplementary Figure 8. (A) Therapy discontinuation for treatment duration subgroup funnel plot (B) Therapy discontinuation for drug type subgroup funnel plot
